# Activation of complement system in kidney after ketoprofen-induced kidney injury in sheep

**DOI:** 10.1186/s13028-015-0106-2

**Published:** 2015-03-15

**Authors:** Mari J Palviainen, Sami Junnikkala, Marja Raekallio, Seppo Meri, Outi Vainio

**Affiliations:** Department of Equine and Small Animal Medicine, Faculty of Veterinary Medicine, University of Helsinki, P. O. Box 56, FI-00014 Helsinki, Finland; Department of Veterinary Biosciences, Faculty of Veterinary Medicine, University of Helsinki, P.O. Box 66, FI-00014 Helsinki, Finland; Department of Bacteriology and Immunology, Haartman Institute, University of Helsinki, P.O. Box 21, FI-00014 Helsinki, Finland

**Keywords:** Acute kidney injury, Complement, Ketoprofen

## Abstract

**Background:**

Non-steroidal anti-inflammatory drugs (NSAIDs) are widely used to treat inflammatory pain in humans and animals. An overdose of an NSAID is nephrotoxic and can lead to acute kidney injury (AKI). Complement activation occurs in several types of renal disorders with proteinuria. The aim of this study was to investigate whether complement system becomes activated in kidneys after a high dose of NSAID. Kidney tissue and urine samples were collected from six sheep with ketoprofen-induced AKI and from six healthy control sheep. The localization of complement proteins in kidney tissue was carried out using immunohistochemical stainings, and excretion of C3 was tested by immunoblotting.

**Results:**

The complement system was found to become activated in the kidney tissue as demonstrated by positive immunostaining for C1q, C3c, C4c, C5, C9 and factor H and by Western blotting analysis of C3 activation products in urine samples in sheep with AKI.

**Conclusions:**

Our results thus suggest that the alternative complement pathway is activated, and it may contribute to the acute tubular injury seen in the kidneys of NSAID-induced AKI sheep. Inhibition of complement activation may serve as potential therapeutic target for intervention in drug-induced AKI.

## Background

The complement system is composed of a large set of soluble and membrane-bound proteins, which act to mediate inflammation, tissue clearance of dying cells, and protection against infection [1,2]. Although the majority of circulating complement C3, the main complement component, is synthesized in the liver, the kidney also contributes to the systemic pool of C3 [3]. Small amounts of complement component C3 are activated in uninjured kidney but complement regulatory proteins, such as factor H, MCP (CD46) and DAF (CD55), inhibit any further activation of the complement cascade [4-8]. In rodents, Crry (complement receptor 1-related protein y) serves the same role as both human regulatory proteins CD46 and CD55 [9,10]. Complement has been shown to be under a state of continuous, but low-level activation via the alternative pathway. The proximal tubular cells seem to be involved in the activation of this alternative pathway in the kidneys [11]. Complement activation is associated with a large variety of renal disorders with proteinuria both in humans and animals. These include various forms of glomerulonephritis [12], renal infarction [13], ischemia-reperfusion injury [14,15] and IgA nephropathy [16].

Nonsteroidal anti-inflammatory drugs (NSAIDs) are widely used to treat acute and chronic inflammatory conditions and pain in humans and animals. NSAIDs block vasodilatory prostaglandins by inhibiting cyclo-oxygenase enzymes (COX-1 and COX-2) that are essential for prostaglandin synthesis [17]. Both COX-1 and COX-2 are expressed in the kidneys of mammals, although some differences between species occur in the localization of the mature proteins [18]. COX-1 is constitutively expressed in most mammalian tissues and it is the most abundant COX isoform in the kidney [19-21]. In contrast, COX-2 exists only at low levels in normal tissues including the kidney. However, COX-2 levels are increased in response to injury or inflammation [22,18]. Prostaglandins (PGs) synthesized in the kidneys modulate renal blood flow, glomerular filtration rate in addition to facilitating urinary sodium and water excretion [23,24]. The use of NSAIDs has been reported to increase the risk of acute kidney injury in humans [25-28], cats and dogs [29-31]. Since complement activation is associated with different renal disorders with proteinuria, we wanted to investigate the possible activation of the complement system in ketoprofen-induced acute kidney injury in sheep.

## Methods

### Animals

Urine and tissue samples were collected from 12 female Finnish Landrace sheep. All sheep were >18-months-old and their body weights ranged from 48.5 to 59 kg (mean 52.5 kg). No clinical signs of diseases were detected in any of the sheep before the trial, and no changes indicative of renal disease were detected in their plasma or urine samples. The sheep were provided with good quality hay and water *ad libitum* before and during the experiment. Six of the 12 sheep were administered a high dose of ketoprofen intravenously (30 mg/kg), the other six sheep did not receive any injections. The study protocol was approved by the Ethics Committee for Animal Experimentation at the University of Helsinki.

### Sample collection

Blood samples were collected into tubes containing lithium heparin before treatment (time 0) and at 1, 2, 4, 6, 8, and 24 hours after ketoprofen administration. Urine specimens were collected from the six sheep with acute kidney injury and their controls before treatment and at two, four, and six hours after ketoprofen administration via a urinary catheter (All-Silicone Foley Balloon Catheter, Sewoon Medical Co). At the end of the study (24 h after treatment), the treatment animals and their controls were euthanized and the last urine samples were collected via cystocentesis and autopsies were performed. AKI was confirmed later by increased plasma urea and creatinine concentrations, proteinuria, enzymuria (Table 1) and histopathology indicative of acute tubular injury (ATI) as described in our previous report [32]. Tissue samples from kidneys were collected immediately after euthanasia and snap-frozen in liquid nitrogen and stored in −80°C freezer for later preparation and analysis.

**Table 1:** **Median (range) values for the plasma and urine variables in samples collected from six sheep with ketoprofen induced AKI confirming renal impairment and from six control sheep** [32]

		**Plasma**		**Urine**				
		**Creatinine μmol/L**	**Urea mmol/L**	**Creatinine μmol/L**	**Prot:crea ratio**	**ALP:crea ratio**	**GGT:crea ratio**	**NAG:crea ratio**
0 h	case	86 (80–106)	5.3 (3.9-6.9)	17.8 (8.0-21.8)	0.11 (0.09-0.18)	3.2 (1.9-5.0)	0.86 (0.50-1.18)	0.03 (0.02-0.04)
	contr	92 (81–113)	4.7 (3.5-5.2)	12.4 (8.9-14.9)	0.14 (0.08-0.38)	3.7 (2.6-7.4)	0.94 (0.56-1.14)	0.03 (0.02-0.04)
2 h	case	89 (82–109)	5.8† (4.5-7.6)	10.9 (3.7-16.6)	0.96† (0.56-1.5)	4.3 (1.9-9.7)	0.86 (0.79-1.38)	0.12*† (0.04-0.26)
	contr	89 (79–106)	4.9 (3.6-6.1)	13.4 (10.9-25.4)	0.49† (0.18-1.38)	4.3 (1.6-10.6)	0.81 (0.39-1.98)	0.02 (0.02-0.03)
4 h	case	120*† (100–140)	7.2*† (5.6-8.8)	7.2*† (4.5-10.0)	7.72*† (4.09-19.6)	13.9*† (9.4-42.4)	1.66 (0.49-4.20)	NM
	contr	91 (74–101)	4.6 (3.7-5.7)	14.9 (6.3-27.1)	0.78† (0.34-1.77)	5.2 (1.4-13.2)	1.01 (0.37-2.95)	NM
6 h	case	151*† (131–191)	8.8*† (6.6-9.6)	3.9*† (2.2-7.9)	18.0*† (6.69-63.1)	42.3*† (27.7-135.8)	3.02*† (1.27-8.17)	0.73*† (0.17-1.40)
	contr	90 (71–102)	4.1 (3.4-5.7)	15.3 (12.7-25.6)	1.41† (0.38-2.67)	5.8 (2.5-18.6)	1.07 (0.56-3.69)	0.02 (0.02-0.04)
8 h	case	184*† (150–208)	9.8*† (7.1-10.4)	2.7*† (1.4-6.5)	27.2*† (3.05-112.9)	98.2*† (16.2-420.8)	6.17*† (2.62-17.53)	NM
	contr	88 (74–105)	3.4 (2.8-5.0)	12.8 (6.6-24.8)	1.36† (0.69-3.58)	9.6 (3.0-20.1)	1.34 (0.40-3.35)	NM
24 h	case	390*† (131–414)	20.6*† (14.0-22.3)	2.7† (2.2-3.5)	3.76† (0.97-16.5)	27.4† (8.7-86.3)	2.62 (0.89-16.13)	0.26*† (0.05-0.60)
	contr	90 (71–101)	4.1 (2.6-7.2)	9.1 (2.1-20.0)	0.77† (0.23-3.60)	8.5 (4.4-9.9)	1.33(0.78-2.93)	0.04 (0.02-0.15)

*Within a time point within a variable, value differs significantly (*P* < 0.05) from the value for the control sheep.

†Within a row, value differs significantly (*P* < 0.05) from the value of baseline.

NM Not measured.

### Immunohistochemical staining of kidney tissue

Snap-frozen kidney tissue samples were embedded in OCT compound (Tissue-Tek, Sakura) and cut into 8 μm sections on a cryostat at −20°C. The localization of complement proteins in kidney tissue was carried out using antibodies raised against human complement proteins that cross-react with respective sheep proteins, namely: polyclonal anti-C1q (Dako Cytomation), polyclonal anti-C3c (Behring) reacting with C3, C3b, iC3b and C3c, polyclonal anti-C3d (Dako Cytomation), polyclonal anti-C4c (Dako Cytomation) reacting with C4, C4b and C4c, monoclonal anti-C5 (Quidel), monoclonal anti-C9 (Quidel) and polyclonal anti-factor H (Quidel). The polymer technique (Goat-on-rodent HRP-polymer and Rabbit-on-pharma HRP-polymer, Biocare Medical) and nickel-enhanced DAB chromogen (Biocare Medical) were used according to manufacturer’s instructions to visualize the bound antibodies. Negative control sections were processed in parallel without the primary antibody. Sections were counterstained with Meyer’s hematoxylin (Fisher).

### Western blot analysis of urine

Western blot analysis was conducted on the urine samples. Individual urine samples were concentrated by TCA-precipitation and the concentrations of proteins in the samples were measured by using 2D Quant Kit (GE Healthcare) according to the manufacturer’s instructions. Sodium dodecyl sulphate-polyacrylamide gel electrophoresis (SDS-PAGE) was carried out in 12% polyacrylamide gels using a vertical slab gel apparatus under non-reducing conditions (Bio-Rad TetraCell) as described previously [33]. A total of 5 μg of protein from each sample was loaded into the gel and the proteins were separated by electrophoresis at 100 V for 2 h 30 min. Proteins were transferred to a PVDF membrane (Immobilon, Amersham) using a semi-dry blotting apparatus (Bio-Rad). Nonspecific binding to the membranes was blocked by an incubation for 1 h using 5% bovine serum albumin in Tris-buffered saline containing 0.1% Tween-20 (TBST). The membranes were thereafter probed with polyclonal anti-C3c in TBST at 4°C. Membranes were then washed with TBST and incubated for 3 h at room temperature with alkaline phosphatase-conjugated anti-rabbit (1:2000, Santa Cruz) in TBS. Proteins were visualized using Super Signal West Dura chemiluminescence substrate (Thermo) and imaged by the LAS3000 image analyzer (Fuji).

## Results

**Figure 1:**
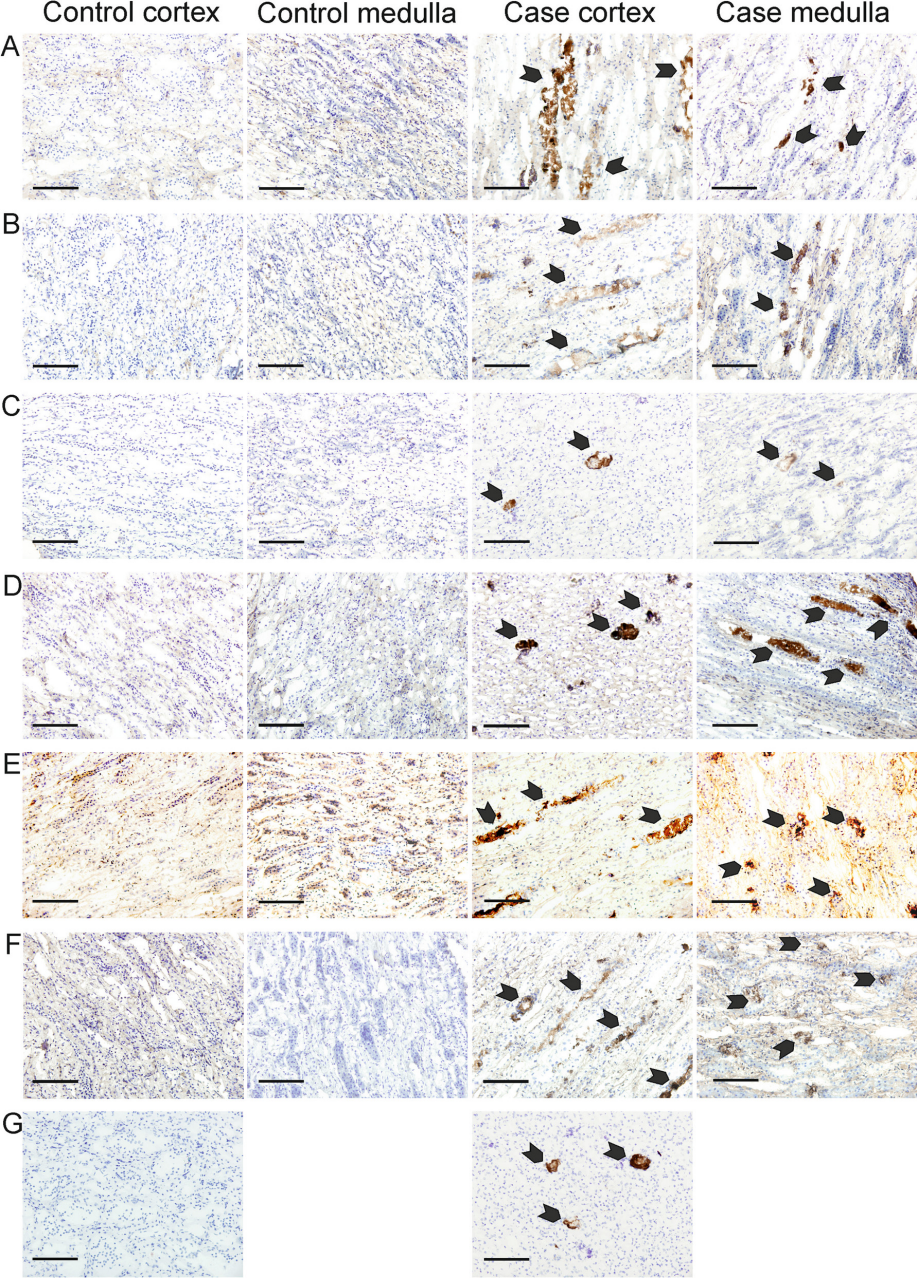
**Immunostaining of control sheep and sheep with ketoprofen-induced AKI renal cortex and medulla using antibodies against A) C1q; B) C3c; C) C3d; D) C4c; E) C5; F) C9; G) factor H.** The arrows indicate the positively stained complement components **(A-G)** in the cortex and medulla of sheep after NSAID-induced AKI. Original magnification obj. 20 X.

Immunohistochemical staining of the normal kidney tissue (control sheep), revealed that the basement membranes of blood vessels, epithelial cells in the tubuli and Bowman’s capsule in the glomeruli were positively stained for C3. The tubulointerstitium in the medulla showed positive staining for C3d. For complement C4, normal kidney (control sheep) showed positive staining in the distal convoluted tubules and in the proximal tubular epithelia in the cortex. In normal kidneys (controls) C5 and C9 were present in the proximal tubular epithelial cells in the medulla. C1q and factor H were absent from the normal kidney tissue (Figure 1).

In the AKI kidney, the deposition of C3 was more intense in the epithelial cells of the proximal tubules of medulla and in the tubular lumina than in those of the controls. However, the AKI kidney showed positive staining for C4 in the distal convoluted tubules and in the proximal tubular epithelia in the cortex, similar to those of the healthy control tissue. Staining also occurred in the proximal tubuli in the medulla, where C4 localized into the epithelial cells and associated with cellular debris in the tubular lumina. C3d showed positive staining in the proximal tubular epithelial cells and in the tubular lumen in the medulla of all the AKI sheep. Moreover, C3d was found in the distal convoluted tubules in the cortex in two of the sheep with AKI. C1q was also found in the proximal tubuli in medulla and in the tubular lumina after AKI. C5 and C9 stained positive in the distal convoluted tubules and proximal tubular epithelia, and in the tubular lumina, intensifying from the cortex to the medulla. Factor H showed strong positive staining in the proximal tubule in the inner medulla of all affected sheep. Two sheep with AKI also stained positive for factor H in the proximal tubuli in the outer medulla and distal convoluted tubuli in the cortical area (Figure 1).

**Figure 2:**
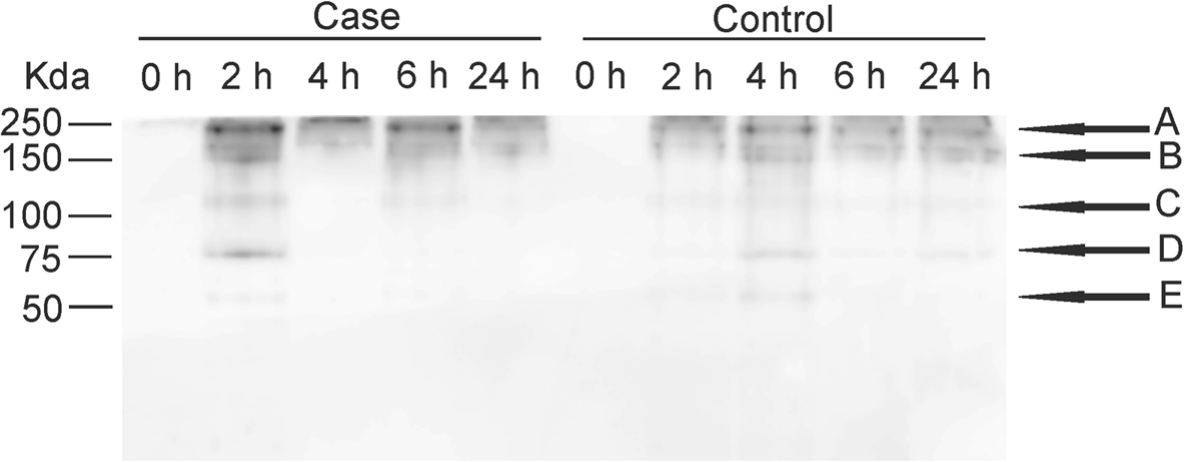
**A representative image of Western blot analysis of the urine of control and ketoprofen-induced AKI sheep.** The proteins were separated on 12% SDS-PAGE in non-reducing condition and they were detected using polyclonal anti-C3c. The observed bands were: **A)** C3; **B)** C3b and iC3b; **C)** C3c; **D)** C3α´75 kDa; **E)** C3dg.

Western blot analysis of urines revealed C3 excretion in both groups (Figure 2). An antibody, which was raised against C3c for the Western blot analysis detected one protein band at the 0 h time point and multiple protein bands in the later samples. The excretion patterns were similar for both AKI and control sheep groups showing intact C3 (~190 kDa) and its activation C3b (~180 kDa) and C3b inactivation products iC3b (170 kDa), C3c (~140 kDa) and C3dg (~40 kDa).

## Discussion

As reported in our previous publication and further shown in Table 1, proteinuria was detected in sheep after ketoprofen induced AKI by an elevated urine protein to creatinine ratio [32]. Proteinuria is usually a consequence of protein leakage in the glomeruli that leads to tubular injury [34,35]. It is believed that the tubular epithelium plays a critical role in the inflammatory and fibrotic changes seen in the interstitium when exposed to high concentrations of protein [36]. Proteinuria also increases tubular cellular turnover. This, in turn, leads to the development of tubular atrophy and disturbs the inhibition of the complement system by preventing factor H binding to the proximal tubular cells [37,38]. In the present study positive immunostainings for C3, C4, C1q, C5, C9 and factor H in the kidney tissue, and Western blotting analysis for C3 activation products in the urine demonstrated activation of complement. Our findings are in line with previous reports suggesting that protein leakage in the glomeruli causes renal toxicity via inflammatory and fibrinogenic pathways in the proximal tubular cells [39-42].

As reported earlier, activated C proteins cluster in the epithelium of the proximal tubules during proteinuria in humans [43,11,44,7] and rats [45,46]. In our present study, C activation occurred principally in the proximal tubular epithelial cells and in the tubular lumen. However, C activation occurred also in the distal convoluted tubular epithelial cells to some extent. While intrarenal synthesis of C components could mediate the progression of ketoprofen-induced AKI it is more likely that the majority of complement proteins derive from blood. Activation of the complement system leads to the formation of either soluble (sC5b-9) or membrane associated forms of C5b-9 (membrane attack complex, MAC). MAC contains one molecule of C5b, C6, C7, C8 and multiple molecules of C9 [1], whereas sC5b-9 has only one C9 molecule. MAC adheres onto the cell membrane and induces cell injury and apoptosis [44]. In case MAC complex was formed it could have, at least partly, have been responsible for the cell injury seen in the tubular cells after a high dose of NSAID. Proximal tubular cells have a very low expression of membrane-bound C regulators such as DAF and MCP. The cells can bind factor H; yet excess plasma proteins or low pH can prevent protective effect of factor H [47,38]. The positive immunostaining for factor H in the tubules of all affected sheep suggest that NSAID-induced AKI proteinuria leads to complement activation, C3b deposition and subsequent binding of factor H to C3b. Complement inhibitors, such as eculizumab, may offer more therapeutic options for patients suffering acute kidney injury.

The similarity of the excretion patterns of C3 and its activation products between AKI-sheep and controls suggests that the urinary catheter activated the complement system, probably in the urinary bladder. This bioincompatibility-induced inflammation can occur, when either native or plasma protein-coated surface binds C3 and activates the alternative pathway via a conformational change in the C3 protein allowing the assembly of the alternative pathway C3 convertase [48-50]. The origin of the plasma proteins that bound to the catheter surface in our study could not be ascertained. Urinary proteins originating from plasma or from initial contact with blood during the implantation of a bladder catheter are two possibilities.

## Conclusions

We conclude that the alternative complement pathway is activated, and it may contribute to the acute tubular injury seen in the kidneys of NSAID-induced AKI sheep.
